# Physicians or “Providers?” Inventing Nazi Origins Undermines Debates on Medical Professionalism

**DOI:** 10.1007/s11606-022-07725-9

**Published:** 2022-07-26

**Authors:** Mical Raz, Daniel Freedman, Volker Roelcke

**Affiliations:** 1 University of Rochester, 364 Rush Rhees Library, P.O. Box 270070, Rochester, NY USA; 2 Department of Neurology, Dell Medical School, The University of Texas at Austin, Austin, TX USA; 3 Giessen University, Giessen, Germany

How should we refer to the diverse group of professionals who provide health care? This is a long debate, which has now taken on new form, as recent articles have disingenuously connected the contested term “provider” with the history of Nazi horrors.^1, 2^ As a result, many in the academic medical community are debating whether the term, like some well-known eponyms, needs to be retired. We argue that this is a misunderstanding and misuse of history.

Many physicians do not like the term provider, and they have made this publicly clear over the past four decades. In 1986, Connecticut physician Lee Sataline wrote the *New York Times*, bemoaning the “double speak” of insurance, and lamenting that on insurance forms physicians are referred to as “providers” and the patients as “consumers,” noting it imbued medicine with a “supermarket touch.”^3^ In 1993, conservative columnist William Safire torched the term “managed competition,” at the base of the proposed Clinton health reforms, while criticizing the term provider. The column opened with a quote from a concerned physician who noted he was “distressed” at being called a “health care provider” and tellingly added that it was “easy to regulate providers but more difficult to regulate doctors.”^4^

The term provider, which first appeared in the mid-1960s with the passage of Title XIX of the Social Security Act and the creation of Medicare and Medicaid, reflected the realities of third-party insurance and its regulation. A service is provided, and the provider of services, initially referring to “hospital, extended care facility, or home health agency,” and only later expanded to include health care professionals, received payment by a third party.^3^

In the past decade, multiple perspective pieces on the term “provider” have been published in top journals. These generally argue that the word diminishes the physician-patient relationship, blurs distinctions between various health care professionals, and enshrines a transactional, rather than healing, relationship.^5–9^ Others suggest that replacing the term holds the stakes for the future of medicine, in terms of bolstering “trust in physicians” and helping “rebuild the resilience” needed during the pandemic.^5^ This is not a new argument, but rather one that harkens to over a century of physicians’ concerns about regulating their profession and the perceived risks of third-party payors that may reduce physician autonomy of practice.^10^ Yet it ignores an important historical context.

In fact, the “patient as consumer” movement was fueled by patient activists, who were eager to reclaim power, autonomy, and independence. As historian Nancy Tomes has shown, the 1970s patient-consumer movement was a response to medical paternalism, and an unresponsive medical establishment.^11^ Patient activism was subsumed into a form of consumer activism, which brought about a realignment of forces in the practice of medicine. These activities included women demanding doctors abandon radical mastectomies in favor of lumpectomies, patients advocating for lower-cost medications, and advocacy groups working to articulate various forms of “patient bills of rights.”^12–14^ As critiques have rightfully argued, these small-scale reforms, while important, also served to hinder larger calls for an overhaul of the health care system.^14^ The patient as a consumer, and the physician as a provider, was a result of sustained pressure of patient activists. Ignoring this history means ignoring the advocacy of the very people we serve.

In 2019, in a polemic blog post with the egregious title “If You Call Me a Provider, I Will Assume You are a Nazi,” the author argued that the term provider has Nazi roots.^15^ For this claim, the author relied on the unfortunate mistranslation in a single essay from a decade and a half prior that focused on the plight of Jewish pediatricians under the Nazi regime.^16^ This erroneous argument was uncritically amplified by a number of other online publications and social media posts, and made its way to the mainstream in 2021, appearing in academic articles.^1, 2^ The reference to the supposed Nazi origin of the term “provider” is now used as an argument not only against the use of the term, but also to highlight what some see as an erosion in the status and independence of the physician.

This argument is factually baseless and morally flawed. First, it builds on an erroneous and misleading translation of the German term *Krankenbehandler*, which the Nazi authorities introduced for Jewish physicians in 1938. It was a newly coined concept which had no echoes in contemporary Nazi nor in post–World War II economic contexts, and had no economic parallels or overtones. As such, there is no adequate English translation. A literal translation of the term’s components might be “sick-treater,”—a term devoid of any transactional nature.^17^ This is in stark contrast to the German term *Anbieter* which is in use today as the equivalent to the English “provider” in German debates on health care provision since the 1970s.^18^ Similar to the US context, this term originated in the sphere of economics and consumer rights, and was transferred from this context to the sphere of health care—it has no semantic or historical connections whatsoever to the term *Krankenbehandler*. This history would be readily evident to any student of the Holocaust, or any practitioner familiar with the current German health care system.

Second, this equation of *Krankenbehandler* and “provider” is morally problematic as it completely ignores the context of its introduction in 1938. The intention by the Nazi authorities was not to identify or analogize Jewish physicians with a broader group of health professionals, or any other individual providing services or commodities. Rather, this term helped enshrine a racialized health care system in which the newly created subgroup of Jewish *Krankenbehandler*, or “sick-treaters” was only allowed to care for Jewish patients.

Some physicians in the USA worry that the term “provider” devalues their unique professional skill and expertise in comparison to other colleagues, such as nurse practitioners, certified registered nurse anesthetists (CRNAs), and physician assistants.^1^ The Nazi category of *Krankenbehandler* resulted in a massive restriction of their immediate legal capacity to act as a physician, resulting in delicensure for the vast majority of Jewish physicians.^17^ These things are not the same. The mistaken translation and equation of *Krankenbehandler* to “provider” falsely equates physician concerns over perceived professional devaluation with the systematic persecution and mass murder of Jewish physicians. In a time of rising anti-Semitism and unfortunate politicization of studying the Holocaust, instrumentalizing the history of the Holocaust to revisit this debate over the term provider is harmful.

Physicians who have argued for replacing the term provider acknowledge that this will be an onerous process, requiring “rewording of regulations, policies and electronic health record interfaces.”^5^ They have argued for a concerted effort involving professional societies, engaging government entities, and even private electronic medical efforts, suggesting that such a profound effort is necessary, and absent such “could potentially increase the likelihood of greater dissatisfaction, confusion and expense in the long run.”^5^ After over four decades of physicians’ writing about their concerns over the term provider, perhaps these energies would be better spent elsewhere.

For those physicians who are worried about engendering trust, perhaps they should join forces with other members of the medical community and continue to work on rebuilding trust, particularly with marginalized populations, and to demonstrate that the medical community is worthy of their trust. Trust is lost when physicians use their powerful platform to perpetuate false historical arguments. This is particularly ironic when physicians argue for the unique nature of their profession and expertise, by making erroneous claims outside of their area of professional expertise. Physicians who are worried about resilience, rather than agitating to replace terminology at multiple societal levels, should come together to advocate for better working conditions for trainees, all health care workers, and for our colleagues in underserved and understaffed hospitals. And finally, if physicians are genuinely appalled by the transactional nature of medical care as evidenced by a “market-based” term, perhaps it is time for physicians to actively work to expand and democratize access to high-quality care for our patients, rather than making ahistorical arguments about the origin of the word provider.
